# Towards a pan marsupial sero-immunological tool in the demanding field of wildlife serology: Marsupial immunoglobulin-binding capability with protein A/G, protein L and anti-kangaroo antibody

**DOI:** 10.1371/journal.pone.0295820

**Published:** 2023-12-14

**Authors:** K. L. D. Tharaka D. Liyanage, Paola K. Vaz, Abdul Jabbar, Jasmin Hufschmid

**Affiliations:** 1 Department of Veterinary Biosciences, Melbourne Veterinary School, Faculty of Science, The University of Melbourne, Werribee, Victoria, Australia; 2 Asia-Pacific Centre for Animal Health, Melbourne Veterinary School, Faculty of Science, The University of Melbourne, Parkville, Victoria, Australia; Oswaldo Cruz Institute, BRAZIL

## Abstract

Detection of infections in wildlife species is increasingly important to reduce the risk of spreading zoonotic and economically important parasites, understand disease epidemiology and promote the conservation of wildlife species. Serological tests are key in disease diagnosis and surveillance by detecting immunoglobulins against infectious agents. However, the need for species-specific reagents has limited the application of serological tests in wildlife species. This study evaluated the serum immunoglobulin-binding capability of polyclonal anti-kangaroo antibody and two non-species-specific reagents, including protein A/G and protein L, with the largest range of Australian marsupial species so far, including 32 species representing three major marsupial orders. Immunoglobulin-binding capability was assessed using immunoblotting, enzyme-linked immunosorbent assay and Western blot techniques. Variation in immunoglobulin-binding capability was observed between the three reagents and across the species tested, both across but also within taxonomic groups. Taxonomic distance was thus not always a good predictor of immunoglobulin-binding affinity, emphasizing the need to validate these reagents for each species separately. However, all three reagents bound with the serum immunoglobulins of most marsupial species tested. The findings of this study provide a valuable reference for species differences in affinity to protein A/G, protein L and anti-kangaroo antibody, assisting in the selection of appropriate reagents and the development of sero-immunological assays in Australian marsupials.

## Introduction

In recent decades, Australian marsupial species have been facing many challenges, including habitat loss and degradation, invasive species and disease [1–3]. It is now widely acknowledged that infectious disease can have substantial negative impacts on wildlife populations, including catastrophic population declines such as have been observed in Tasmanian devils (*Sarcophilus harrisii*) with Devil Facial Tumour Disease [4]. Effective disease surveillance and studies into disease epidemiology are required to monitor the population health of marsupials, particularly endangered species. Moreover, understanding the prevalence of infections in marsupials is also important to reduce the risk of infectious agents transmission to animals of economic and health interest and humans, such as through the kangaroo meat industry [5, 6]. However, these approaches are reliant on detailed insights into marsupial immunological responses [7, 8].

Immunological differences between mammalian taxonomic groups can be profound and may be related to evolutionary processes. Mammals evolved around 200 million years ago and are classified into three major subclasses: eutherians (placental mammals), metatherians (marsupials) and prototherians (monotremes) [9, 10]. Prototherians were the first to diverge from their common ancestor around 160 million years ago [11], followed by metatherians who diverged from eutherians around 148 million years ago. There are now over 300 species of marsupials inhabiting the American and Australasian geographic regions [12, 13], two-thirds of which are endemic to Australia. Modern Australian marsupial species are divided into four taxonomic orders: the Dasyuromorphia, Peramelemorphia, Diprotodontia and Notoryctemorphia [14]. This unique Australian marsupial fauna results from the continent’s geographic isolation from other land masses for more than 50 million years, during which marsupials adapted successfully into diverse habitats ranging from deserts to wetlands [14].

There used to be a belief that marsupials had less complex, primitive immune systems compared to their eutherian counterparts [15]. However, marsupials diverged from eutherian mammals after the evolution of immune systems, and therefore both subclasses of mammals have similarly complex immune systems. This includes sharing many similarities in the structure and function of both their innate and adaptive immune responses [8]. The B-cells of eutherian adaptive immune systems produce five immunoglobulin isotypes (IgG, IgM, IgD, IgE and IgA), each possessing different immunological roles [16]. A single immunoglobulin molecule consists of two identical heavy chains and two identical light chains; the heavy chain types (α, γ, δ, ε, μ) are responsible for the classification of each isotype. Light chains are further classified into either kappa (k), or lambda (λ) based on slight variations in their polypeptide sequences [17]. Marsupials produce four of these five immunoglobulin isotypes, including IgG, IgM, IgE and IgA [10]. However, IgD has not been observed in marsupials and a genomic region encoding the IgD heavy chain is absent in marsupial genomes sequenced to date [10, 18].

Serological tests can detect exposure to infectious agents by identifying antigens or pathogen-specific immunoglobulins in serum. One of the major problems in wildlife serological testing is the lack of commercially available species-specific immunoglobulin-binding conjugates [19]. Commercially available conjugates made for domesticated species have been used in closely related wildlife species [20, 21], but they are not always reliable for phylogenetically distant wildlife species [22] and, without proper validation, pose the risk of false negative results. This is primarily due to between-species differences in immunoglobulin-binding capability [22–24]. On the other hand, producing species-specific immunoglobulin-binding agents for use in wildlife is laborious, time-consuming, expensive and requires expert knowledge.

Among the most commonly used non-species-specific immunoglobulin-binding reagents in mammals are protein A, protein G and protein L. Protein A and G are microbial cell wall proteins derived from *Staphylococcus* sp. *and Streptococcus* sp., respectively [25], and capable of binding with the constant (Fc) region of IgG immunoglobulins across a range of mammalian species [26]. These non-species-specific immunoglobulin-binding proteins have been widely used in immunoglobulin purification techniques and serological assays [27, 28]. However, the immunoglobulin-binding capability of these two proteins varies and is also different across species [25, 26]. Vaz et al. [29] investigated the serum immunoglobulin-binding capability of protein A and protein G with 15 Australian marsupial species, representing two marsupial orders, including the Diprotodontia and Dasyuromorphia. They found that the IgG of most of the marsupial species tested bound to protein A, but only a few to protein G. Protein A/G is a recombinant fusion protein produced by the fusion of the Fc-binding domains of both protein A and G [30]. Therefore, protein A/G can bind with the IgG of a broader species range compared to protein A or G alone [31]. Protein A/G has been used in several wildlife serological studies [32, 33], but the binding capability of the protein conjugate A/G has not been evaluated across a wide range of marsupial species to date. Protein L is isolated from the surface of *Peptostreptococcus magnus* [26, 34] and binds specifically with the kappa light chain of immunoglobulins without interfering with antigen binding sites [34]. Since protein L is not heavy chain specific, it can bind with a wide range of immunoglobulin classes [35]. Subsequently, protein L maybe a useful serological tool for detecting total immunoglobulin response, including either or both IgM and IgG, reflecting different stages of infection.

Rabbit polyclonal anti-kangaroo whole serum antibody is the only commercially available anti-marsupial antibody used in serological assays. This reagent has been used across a number of marsupial species to detect immunoglobulins to infectious agents in enzyme-linked immunosorbent assays (ELISA) [5, 36–38]. Vaz et al. [29] showed strong binding of this reagent to eight macropod and seven non-macropod sera. However, the capacity of this reagent to be used in marsupial serological assays should be further extended to confirm its validity across marsupials representing additional species, families and orders.

This study aimed to compare and evaluate the immunoglobulin binding capability of three commercially available reagents (Protein A/G, protein L and anti-kangaroo antibody) across an expanded list of Australian marsupial species, representing families across three orders (Dasyuromorphia, Peramelemorphia and Diprotodontia), and with an increased focus on developing serological diagnostic methods to detect infectious agents among Australian marsupial species.

## Materials and methods

### Acquisition of samples and reagents tested

Serum samples from 32 Australian marsupial species, representing three marsupial orders [Diprotodontia (*n* = 26), Peramelemorphia (*n* = 3) and Dasyuromorphia (*n* = 3)] and five eutherian control species were included in this study (Table 1). Samples of equal volume from up to five individuals were pooled for each species, depending on the availability of serum. All serum samples were stored at -20°C for further use. Serum samples used in this study were from our laboratory serum archives, the Melbourne Zoo archives or a previous study [29]. Samples from Melbourne Zoo were obtained with approval by the Zoos Victoria Animal Ethics Committee (AEC) (ZV21010), and work was conducted with a Department of Environment, Land, Water and Planning (DELWP) Wildlife Research Permit (Permit No: 10010080).

**Table 1:** List of species used to investigate serum immunoglobulin-binding capability of each species against protein A/G, protein L and anti-kangaroo antibody, showing the reciprocal of endpoint serum dilution values above the cut off (OD = 0.1) as measured by ELISA.

No	Order	Family	Species name	Common name	n	Endpoint serum dilution		
						Protein A/G	Protein L	Anti-kangaroo
1	Diprotodontia	Macropodidae	*Macropus dorsalis*	Black-striped wallaby	5	108,990	111,064	1,107,789,893
2	Diprotodontia	Macropodidae	*Macropus parma*	Parma wallaby	5	128,852	120,329	1,128,110,965
3	Diprotodontia	Macropodidae	*Macropus parryi*	Whiptail wallaby	5	141,028	131,287	1,146,399,931
4	Diprotodontia	Macropodidae	*Petrogale lateralis*	Black-flanked rock-wallaby	4	126,595	108,913	1,100,371,206
5	Diprotodontia	Macropodidae	*Petrogale penicillata*	Brush-tailed rock-wallaby	4	139,194	123,943	1,115,758,605
6	Diprotodontia	Macropodidae	*Petrogale xanthopus xanthopus*	Yellow-footed rock-wallaby	5	129,690	91,010	1,133,344,204
7	Diprotodontia	Macropodidae	*Macropus rufogriseus*	Red-necked wallaby	5	131,250	103,092	1,157,757,651
8	Diprotodontia	Macropodidae	*Macropus agilis*	Agile wallaby	2	141,527	124,673	1,120,962,138
9	Diprotodontia	Macropodidae	*Macropus giganteus*	Eastern grey kangaroo	5	125,701	121,385	1,198,641,553
10	Diprotodontia	Macropodidae	*Macropus rufus*	Red kangaroo	5	127,465	79,798	1,147,177,202
11	Diprotodontia	Macropodidae	*Macropus fuliginosus fuliginosus*	Western grey kangaroo	4	133,295	104,783	1,155,460,743
12	Diprotodontia	Macropodidae	*Macropus antilopinus*	Antilopine kangaroo	4	127,465	110,288	1,099,546,840
13	Diprotodontia	Macropodidae	*Dendrolagus goodfellowi*	Goodfellow’s tree-kangaroo	5	95,340	118,577	1,137,586,698
14	Diprotodontia	Macropodidae	*Macropus robustus erubescens*	Common wallaroo	3	120,568	104,856	1,217,416,933
15	Diprotodontia	Macropodidae	*Dorcopsis luctuosa*	Gray dorcopsis	5	122,117	131,412	1,119,846,645
16	Diprotodontia	Potoroidae	*Bettongia penicillata ogilbyi*	Woylie/Brush-tailed bettong	1	98,929	127,326	1,179,862,474
17	Diprotodontia	Potoroidae	*Bettongia lesueur*	Burrowing bettong	5	55,708	115,346	1,115,875,921
18	Diprotodontia	Potoroidae	*Potorous longipes*	Long-footed potoroo	1	143,300	148,624	1,073,953,697
19	Diprotodontia	Potoroidae	*Potorous tridactylus tridactylus*	Long-nosed potoroo	5	126,620	119,923	1,097,432,396
20	Diprotodontia	Petauridae	*Gymnobelideus leadbeateri*	Leadbeater’s possum	3	62,902	89,688	1,020,741,397
21	Diprotodontia	Petauridae	*Petaurus breviceps*	Sugar glider	2	120,735	69,549	925,886,742
22	Diprotodontia	Phalangeridae	*Trichosurus vulpecula*	Common brushtail possum	5	118,293	123,380	1,008,192,292
23	Diprotodontia	Phalangeridae	*Trichosurus cunninghami*	Mountain brushtail possum	5	81,299	113,206	1,024,120,246
24	Diprotodontia	Vombatidae	*Lasiorhinus latifrons*	Southern hairy-nosed wombat	5	<50	68,120	1,071,904,106
25	Diprotodontia	Pseudocheiridae	*Pseudocheirus peregrinus*	Common ringtail possum	2	<50	41,022	931,695,447
26	Diprotodontia	Phascolarctidae	*Phascolarctos cinereus*	Koala	5	<50	99,382	1,014,115,551
27	Peramelemorphia	Peramelidae	*Perameles gunnii*	Eastern barred bandicoot	5	<50	87,926	916,902,095
28	Peramelemorphia	Peramelidae	*Isoodon obesulus*	Southern brown bandicoot	5	<50	82,828	928,514,077
29	Peramelemorphia	Thylacomyidae	*Macrotis lagotis*	Greater bilby	4	<50	<50	863,708,059
30	Dasyuromorphia	Dasyuridae	*Dasyurus viverrinus*	Eastern quoll	5	82,386	51,664	1,012,761,901
31	Dasyuromorphia	Dasyuridae	*Dasyurus hallucatus*	Northern quoll	2	20,074	<50	1,027,973,889
32	Dasyuromorphia	Dasyuridae	*Sarcophilus harrisii*	Tasmanian devil	5	114,151	116,314	1,070,138,642
33	Carnivora	Felidae	*Felis catus*	Cat	5	138,849	106,100	<50
34	Artiodactyla	Bovidae	*Bos taurus*	Cattle	5	129,025	<50	<50
35	Galliformes	Phasianidae	*Gallus gallus*	Chicken	5	<50	<50	<50
36	Rodentia	Muridae	*Mus musculus*	Mouse	5	68,935	107,316	<50
37	Perissodactyla	Equidae	*Equus ferus*	Horse	2	121,436	<50	<50

n = number of individual serum samples pooled per species tested.

Sera were evaluated against three commercially available immunoglobulin-binding reagents, including Horseradish Peroxidase (HRP)-conjugated protein A/G (#32490, Thermo Fisher Scientific, Massachusetts, USA), HRP-conjugated protein L (#32420, Thermo Fisher Scientific), rabbit polyclonal anti-kangaroo whole serum antibody (#A140-105, Bethyl Laboratories, Texas, USA). Goat polyclonal anti-rabbit IgG-antibody (#A120-201A, Bethyl Laboratories) was used as the detection antibody in assays using rabbit polyclonal anti-kangaroo whole serum antibody. Optimum working dilutions for each reagent were determined following manufacturer’s guidelines.

### Immunoblots

Immunoblots were initially performed to qualitatively measure and compare the relative immunoglobulin-binding capability among three reagents across the species listed. The immunoblotting procedure was performed as previously described by Vaz [29], with some modifications. Briefly, two-fold serial dilutions from 1:50 to 1:800 were made for each species diluting the sera in phosphate-buffered saline (PBS). Polyvinylidene fluoride (PVDF, Immobilon-P membrane, Merck, Darmstadt, Germany) membranes were used for immunoblotting and the membrane was activated following manufacturer’s instructions. Following activation of the membrane, 4 μl of sera from serial dilutions of each species were spotted in the predetermined locations of the membrane. Subsequently, the membrane was immersed in methanol for 15 seconds and airdried for 15 minutes on PBS-moistened blotting paper to prevent complete drying of the membrane. HRP conjugated protein A/G, HRP conjugated protein L and rabbit polyclonal anti-kangaroo whole serum antibody were prepared in PBS containing 0.05% Tween 20 (PBS-T) and 5% w/v skim milk powder in 1:5000, 1:4000 and 1:4000 dilutions, respectively. Subsequently, membranes were incubated in each immunoglobulin-binding reagent for 1 hour at room temperature on a rocking platform with gentle agitation. Membranes incubated with anti-kangaroo whole serum antibody were incubated with goat polyclonal anti-rabbit IgG-antibody in 1:5000 dilution as described above. Membranes were washed four times with PBS-T for 20 minutes using a plate shaker in-between and after the incubation steps. Washed membranes were then incubated with Clarity Western ECL Substrate (Bio-Rad, California, USA) for 5 minutes at room temperature. Finally, membranes were visualized using ChemiDoc MP Imaging system (Bio-Rad). Level of affinity between immunoglobulin-binding reagents and serum immunoglobulins was determined by the color intensity of the blot, with darker color blots representing stronger binding.

### ELISAs

The ELISAs were performed to quantify the immunoglobulin-binding capability of the three reagents across the listed species. ELISA plates (Nunc MaxiSorp, Thermo Fisher Scientific) were coated with 50 μl of half-log serial dilutions (1:50 to 1:157,318) of sera from each species made in carbonate bicarbonate buffer (0.032 M Na2CO3,0.068 M NaHCO3, pH 9.6). Serial dilutions were further extended up to 1:1,564,142,237 for the assays involving anti-kangaroo antibodies. Mouse serum and coating buffer were included in each ELISA plate to act as internal control and negative control, respectively. Sera of each species were coated as duplicates. Coated ELISA plates were then incubated overnight at 4°C. On the following day, coated ELISA plates were washed three times with PBS-T to remove any unbound material. Then, any free remaining binding sites on the ELISA plate were blocked with 100μl PBS-T (pH 7.4) containing 5% w/v skim milk powder and 10% w/v bovine serum albumin (BSA) fraction-V (Sigma-Aldrich, Missouri, USA) for two hours at 37°C. Following washing three times with PBS-T, plates were incubated for 60 minutes at room temperature after adding 50 μl of HRP conjugated protein A/G (1:10,000) or HRP conjugated protein L (1:2000) or rabbit polyclonal anti-kangaroo whole serum antibody (1:4000) in ELISA diluent (PBS-T containing 2.5% w/v BSA fraction V and 2.5% w/v skim milk powder) to each well of the ELISA plate. The plate was then washed three times with PBS-T, and plates incubated with rabbit polyclonal anti-kangaroo whole serum antibody were then incubated with goat polyclonal anti-rabbit IgG-antibody (1:10,000) in ELISA diluent for 1 hour at room temperature. Subsequently, plates were washed three times with PBS-T. Then, 50 μl ABTS peroxidase substrate (KPL, SeraCare, Massachusetts, USA) was added into each well of the ELISA plate and incubated for 15 minutes at room temperature. The absorbance value (OD) of each plate was read at 405 nM using a microplate reader (MULTISKAN FC, Thermo Fisher Scientific). The mean OD value for each duplicate was calculated. Linear regression analysis (Minitab version 20) of mean OD values was used to determine the highest serum dilution for each species capable of binding with each immunoglobulin-binding reagent. The cut-off OD value to determine the endpoint serum dilution used in this study was 0.1.

### Western blots

Western blots were performed to confirm that each immunoglobulin-binding reagent is binding with the expected size of the immunoglobulins present in the sera of selected wildlife species (Table 1). Western blots were performed under reducing and non-denaturing conditions to preserve the tertiary structure and the function of the immunoglobulins. Briefly, serum samples from each species were diluted 1:25 in native sample buffer (Bio-Rad). Then, 10 μl of diluted serum samples from each species were transferred to the 4–15% Mini PROTEAN TGX precast gel (BioRad). Gels were run at 120 V for 72 minutes at 25°C in the running buffer containing 0.1% sodium dodecyl sulfate (SDS). Proteins were then transferred into the polyvinylidene difluoride (PVDF) membranes (Merck) using the Trans-Blot Turbo transfer system (BioRad) following the manufacturer’s instructions. Then, PVDF membranes were blocked in 5% skim milk powder in PBS overnight at 4°C. Membranes were washed twice, 10 minutes each with PBS-T and incubated with each antibody binding reagent and visualized as described in immunoblots. Furthermore, serum samples from all the species listed were run in 4–15% Mini PROTEAN TGX precast gel (BioRad) following the above-mentioned conditions and stained with Coomassie brilliant blue stain (BioRad) to detect total serum protein profiles.

## Results

A summarized comparison of marsupial immunoglobulin binding affinity to protein A/G, protein L and anti-kangaroo antibody using immunoblot, ELISA and Western blot are shown in S1 Table.

### Immunoblots

**Fig 1:**
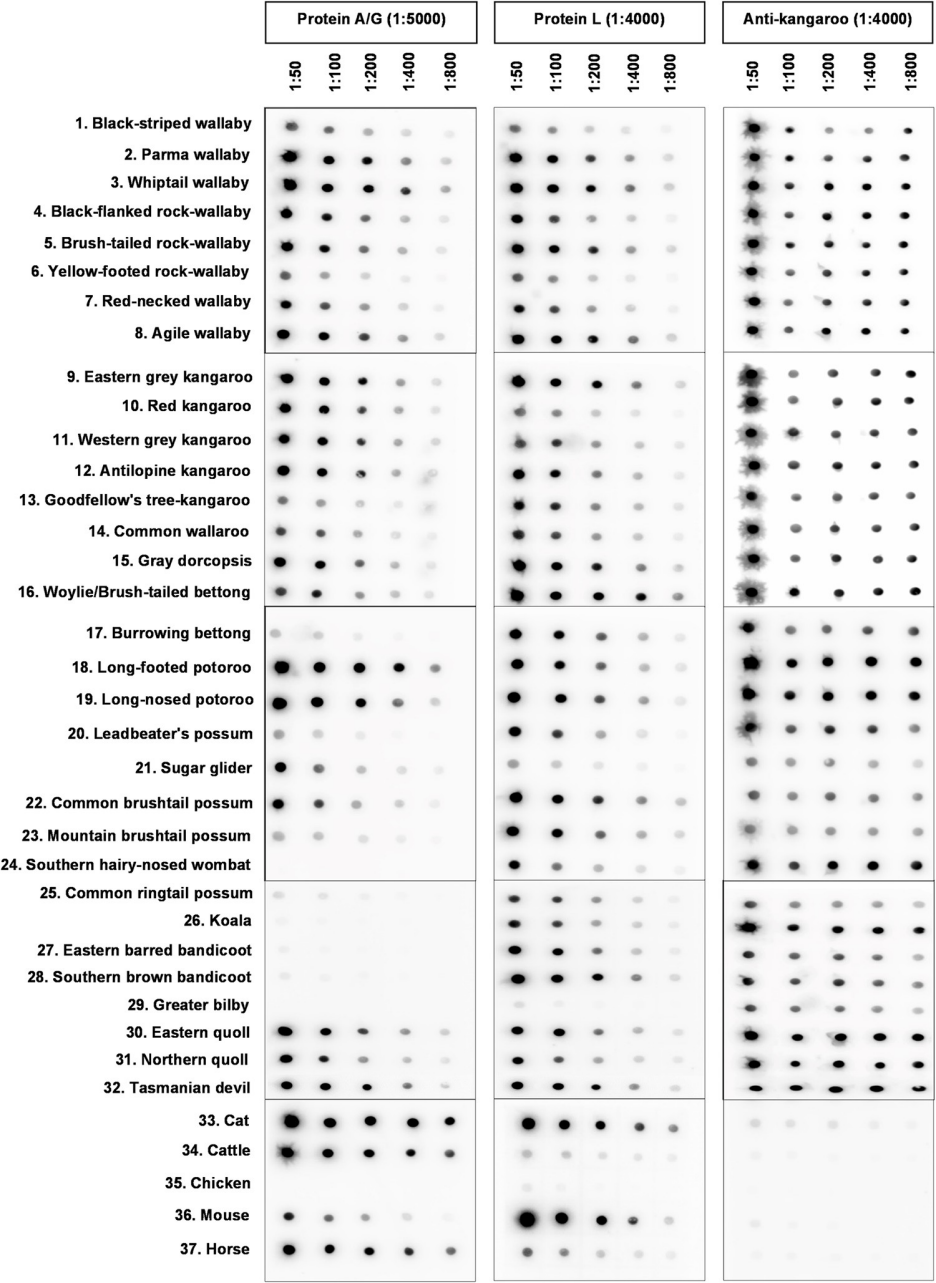
Immunoblot results showing strength of binding to sera of a range of metatherian (marsupial) and eutherian (placental) mammals and non-species-specific immunoglobulin-binding reagents, including protein A/G, protein L and anti-kangaroo antibody. Dilutions are indicated in the top row. Darker colour represents stronger binding.

Visual assessment of immunoblot results confirmed varying degrees of immunoglobulin-binding capability between the three reagents and across the different species tested (Fig 1). All Dasyuromorphia sera appeared to be strongly bound by protein A/G. Protein A/G also seemed to bind strongly with the sera of most macropods (order Diprodontia). However, binding to protein A/G appeared weak to medium across non-macropod members of that order except for long-footed potoroo (*Potorous longipes*) and long-nosed potoroo (*P*. *tridactylus*), for which binding was strong. Sera of common ringtail possum (*Pseudocheirus peregrinus*), Leadbeater’s possum (*Gymnobelideus leadbeateri*), mountain brushtail possum (*Trichosurus cunninghami*), burrowing bettong (*Bettongia lesueur*), koala (*Phascolarctos cinereus*) and southern hairy-nosed wombat (*Lasiorhinus latifrons*) bound at very weak or at undetectable levels with protein A/G at any dilution tested. Binding to protein A/G by members of the order Peramelemorphia, including eastern barred bandicoot (*Perameles gunnii*), southern brown bandicoot (*Isoodon obesulus*) and greater bilby (*Macrotis lagotis*), was very weak to undetectable. Chicken (*Gallus gallus*) serum appeared to be not bound by protein A/G, but the sera of eutherian mammals, including cat (*Felis catus*), cattle (*Bos taurus*), horse (*Equus ferus*), and mouse (*Mus musculus*) to a lesser degree, seemed to bind strongly.

Good to strong binding to Protein L was observed in the sera of 28 marsupial species tested, across all three orders; however, weak binding was seen with sugar glider (*Petaurus breviceps*) (Diprotodontia) and greater bilby (Peramelemorphia) sera. Cat and mouse sera seemed to bind more strongly with protein L than horse and cattle sera, and there was very weak to no binding with chicken sera. Anti-kangaroo antibody showed very strong binding to the sera of most of the marsupial species tested, but only very weak to no binding with the sera of eutherian and avian species.

### ELISAs

End point serum dilution values for each marsupial and control species were measured for protein A/G, protein L and anti-kangaroo antibody using ELISAs. The reciprocal values of the end point serum dilution for each species against each of the antibody binding reagents are shown in Table 1.

The ELISA confirmed that the sera of 13 members of Diprotodontia bound strongly to protein A/G; this was especially true for members of the family Macropodidae (1:95,340 to 1:141,527). However, some of the Diprotodontia showed lower endpoint values, such as burrowing bettong and Leadbeater’s possum sera, which bound with A/G at end point dilution values of 1:55,708 and 1:62,902, respectively. Sera from three members of the Diprotodontia, including southern hairy-nosed wombat, common ringtail possum and koala, were not bound by protein A/G at all (all <1:50). None of the tested sera of members of the Peramelemorphia were bound by protein A/G (<1:50). Although sera of all three members of the order Dasyuromorphia were bound by protein A/G (1:20,074 to 1:114,151), they did so with a lower end point dilution in northern quoll (*Dasyurus hallucatus*) (1:20,074). All eutherian (1:68,935 to 1:138,849), but not avian (<1:50), control species were bound by protein A/G.

Protein L showed binding to the sera of all the members of the order Diprotodontia (endpoint dilution values of 1:41,022 to 1:148,624). The sera of most Peramelemorphia and Dasyuromorphia were bound by protein L (1:51,664 to 1:116,314), except for the greater bilby (<1:50) and northern quoll (<1:50). Among control sera, only cat and mouse sera were bound by protein L (1:106,100 to 1:107,316) whereas cattle, chicken and horse sera were not bound by protein L (<1:50).

Anti-kangaroo antibody bound very strongly with sera of all marsupial species at higher end point dilutions (1: 863,708,059 to 1:1,198, 641,553). None of the eutherian or avian control sera were bound by anti-kangaroo antibody (<1:50).

### Western blots

Western blots were performed for the sera of each marsupial species under reducing and non-denaturing conditions. Results obtained from western blots for protein A/G, protein L and anti-kangaroo antibody and gel images of total serum proteins for each species are shown in S1 Fig.

Protein A/G reacted with large proteins of an approximate size of 75–170 kDa, which is consistent with the expected size of intact mammalian IgG immunoglobulins. The reactivity of protein A/G with serum immunoglobulins varied across the species tested. Protein A/G bound in a good to very strong manner with serum immunoglobulins of all members of the family Macropodidae; however, very weak to no reactivity was seen from many other members of the order Diprotodontia, including burrowing bettong, Leadbeater’s possum, sugar glider, southern hairy-nosed wombat, common ringtail possum and koala. Faint bands, indicating weak reactivity with protein A/G, were observed with the serum immunoglobulins of common brushtail possum (*Trichosurus vulpecula*) and mountain brushtail possum. Very weak to no reactivity was observed between Protein A/G and immunoglobulins of all the members of the orders Peramelemorphia and Dasyuromorphia. On the other hand, protein A/G reacted very strongly with the serum immunoglobulins of cat, cattle and horse and moderately with those of mouse. No reaction was observed between protein A/G and chicken serum immunoglobulins.

Protein L reacted with large serum proteins with an approximate size of 75-170kDa in most marsupial species, though with varying degrees of reactivity among species. The serum immunoglobulins of members of the order Diprotodontia varied from weak to very strong reactivity, except sugar gliders, which displayed no reactivity. Among the Peramelemorphia, only eastern barred and southern brown bandicoot serum immunoglobulins were reacted by protein L. Tasmanian devil (*Sarcophilus harrisii*) serum immunoglobulins were the only representatives of the Dasyuromorphia that showed a detectable level of reactivity by protein L. Serum immunoglobulins of all eutherian mammals, but not chicken, reacted by protein L.

Anti-kangaroo antibody was broadly reactive with a range of both large and small serum proteins (15-250kDa) in all marsupial species tested. However, anti-kangaroo antibody showed less reactivity to serum proteins of sugar glider compared to the other marsupial species tested. The serum proteins of eutherian species reacted only weakly with anti-kangaroo antibody. Anti-kangaroo antibody didn’t react with chicken serum proteins.

## Discussion

We evaluated three commercially available reagents for their immunoglobulin-binding capability to sera from 32 marsupial species. Variations in immunoglobulin-binding capability were observed among species tested, reagents and test methods.

The results of the present study substantiate that there are significant differences in immunoglobulin-binding affinity to protein A/G between marsupial species in different, but sometimes also the same, taxonomic groups. For example, protein A/G appears to be a suitable reagent for serological assays in macropods, but, confirming results from Vaz et al. [29], it was found that protein A and G were less suitable for some other species in the same order (Diprotodontia); similar to the present study, they found that koala serum immunoglobulins were weakly bound by protein G and not at all by protein A. In some cases, there were differences at the family level. The lack of binding to protein A/G for the southern hairy-nosed wombats in the present study contrasted with the findings by Vaz et al. [29], who found that bare-nosed wombats (*Vombatus ursinus*) bound strongly to protein A. Subsequently, while none of the serum immunoglobulins of the three members of the order Peramelemorphia tested (eastern barred bandicoot, southern brown bandicoot and greater bilby) were bound by protein A/G, suggesting that serological assays using protein A/G in these species is likely to result in significant under-detection, protein A/G binding capability across other members of that order is unknown and should be specifically investigated. Similarly, comparison of protein A/G binding capability in all three living species of wombat, including bare-nosed wombat, and the con-generic southern and northern hairy-nosed wombat (*Lasiorhinus krefftii*) would be useful. Evolutionary distance is likely to play a significant role for inter-species variation in binding patterns with protein A/G in marsupials due to variation of protein A/G binding domains present in immunoglobulins [31, 39]. Similar differences along taxonomic lines are observed between eutherian mammal species [31, 40]. However, the results presented here show that taxonomic distance is not always a reliable predictor of immunoglobulin-binding affinity. Therefore, it is important to assess the species-specific binding capability of protein A/G before developing serological assays [40] or use any commercially available kits using protein A/G as a conjugate, particularly with wildlife species where limited affinity data is available.

Except for greater bilby and northern quoll, protein L showed good binding with serum immunoglobulins of most marsupial species tested using immunoblots and ELISAs, indicating protein L as a good candidate for many marsupial serological assays. In contrast to protein A/G, which only binds to the Fc region of mammalian IgG, protein L binds with the kappa light chains of any immunoglobulin isotype [30, 34]. Kappa (κ) to lambda (λ) light chain ratios can vary across different eutherian mammalian species. For instance, the κ:λ ratio in mice is 95:5, in horses 10:90 and lambda chains are predominant in cattle [41]. Kappa light chains do not appear to be present in birds [42]. This variation in kappa light chain expression explains the different protein L binding capability observed across the eutherian control species used in this study. The presence of kappa light chains has only been investigated in a few Australian marsupials [10, 41], and the κ:λ ratios in these species are unknown. Nevertheless, the good binding affinity of protein L observed in the present study could suggest better kappa light chain expression and higher κ:λ ratio in many of the marsupial species tested. However, further studies are required for the accurate investigation of kappa and lambda light chain expression and ratios in Australian marsupial species [41] for the development and advancement of immunological tools. While the use of protein A/G limits the application of serological tests to the detection of IgG, which is predominant in the later phase of infection [43], the ability of protein L to bind with a range of serum immunoglobulins, including both IgM and IgG, could be useful in developing a single serological assay to detect both early and later stages of infection, respectively. Such assays are particularly important when surveying animals susceptible to peracutely or acutely succumbing to a disease before the onset of production of IgG. One such example is acute *Toxoplasma gondii* infection in eastern barred bandicoots (*Perameles gunnii*), a species thought to be susceptible to sudden death in the early stages of infection [44], potentially resulting in under-detection by conventional assays testing for IgG, such as the widely used modified agglutination test (MAT).

Due to its wide range of reactivity across many marsupial species, anti-kangaroo antibody appears to be a useful reagent across a range of marsupial serological assays. In the Western blots, anti-kangaroo antibody was broadly reactive with serum proteins of the expected size of immunoglobulins, but also with those smaller and larger. This may, however, not be a significant issue for diagnostic assays such as the indirect ELISA, which capture pathogen-specific immunoglobulins before washing away other proteins in the test sera and the application of the secondary immunoglobulin-binding reagent [43]. Generally, anti-kangaroo antibody is less broadly reactive in an environment where no other serum constituents are present except antigen-bound pathogen specific immunoglobulins.

Immunoglobulin-binding capability across species for each reagent was generally similar between immunoblot and ELISA results. Western blot results (S1 Fig) also aligned with the other tests in most cases, though there were some exceptions. For instance, sera of all three dasyurid species, including eastern quoll, northern quoll and Tasmanian devil, were reacted by protein A/G in dot blots and ELISAs, but weak to no binding was observed in Western blots. No attempt was made to purify immunoglobulins from serum samples, and therefore the possibility of reagents binding with components other than immunoglobulins in the serum cannot be fully excluded. This is especially true for the immunoblot and ELISA assays, which cannot distinguish binding with immunoglobulins from binding with other proteins. However, such non-specific binding seems unlikely, given that specificity to bind with immunoglobulins by protein A/G and protein L has been well documented in eutherian counterparts [31, 45]. Nonetheless, due to the limited studies on seroimmunology and immunogenetics in marsupials compared to eutherians, the probability of yet undiscovered immunoglobulins or other serum proteins in marsupial species capable of reacting with immunoglobulin-binding reagents is not known. Further studies are recommended to better characterization of interactions between microbially derived broad-spectrum immunoglobulin-binding reagents and marsupial serum constituents. The more likely reason for the contrasting findings between test modalities could be changes in the structure and/or immunochemical reactivity of immunoglobulins due to the exposure to low levels of SDS in the running buffer [46]. While exposure to low levels of SDS is known to cause minor changes to function and conformation of eutherian immunoglobulins [47], the extent of the effect of SDS on immunoglobulins of different marsupial species is currently unknown. Moreover, different test formats may produce variable results due to the inherent properties of each test method, hence suitable test formats and reagents should be selected based on the objective of the experiment.

There was no information on disease history or age for most of the samples used for this study, and total antibody levels were unknown. Different immunoglobulin classes are present in varying amounts in the serum [48]. IgG is the most predominant class of immunoglobulin present in the serum, comprising approximately 80% of total immunoglobulins [49]. The level of serum immunoglobulins present in each individual can vary due to several factors, including level of exposure to infectious agents, age and status of the immune system [48, 50]. Therefore, individual intra-species variation in immunoglobulin-binding capability could also lead to an altered interpretation of the final immunoglobulin-binding capability results [31]. Similarly, any degradation of serum immunoglobulins in older archived samples, and the presence of inhibitors [31], could also contribute to the alteration of immunoglobulin-binding capability. To minimize these effects, serum samples from different individuals per each species were pooled for this study. Gel images of total serum protein profiles (S1 Fig) of each species confirmed that each species had visible bands at the expected size of IgG and the presence of other serum proteins.

## Conclusion

Three commercially available reagents, including protein A/G, protein L and anti-kangaroo antibody, were evaluated for their serum immunoglobulin-binding capability across the sera of a range of marsupial species. Variation in immunoglobulin-binding was observed between reagents and between marsupial orders and families, suggesting evolutionary distance may not always be a reliable predictor of antibody binding affinity in marsupials. Therefore, prior validation of these reagents before application in marsupial serological assays is recommended. Similarly, distinctive properties of each reagent indicated their importance in different serological assays and provide a better solution to overcome the lack of species-specific conjugates for Australian marsupial species. The findings of this study may provide valuable information to assess the suitability of these reagents for marsupial serological studies and test development. Further research including more marsupial species, novel non-species-specific reagents [51] and application of these non-species specific reagents in pathogen specific serological assays is recommended to improve serological studies of Australian marsupials.

## Supporting information

S1 Table Comparison of marsupial immunoglobulin binding affinity to protein A/G, protein L and polyclonal anti-kangaroo antibody using immunoblot, enzyme linked immunosorbent assay (ELISA) and Western blot. -, No binding; +, weak binding; ++, moderate; +++, good binding; ++++, strong binding; +++++, very strong binding. Note: Reciprocal of the ELISA OD values were graded as following <50, no binding; 50–75,000, weak; 75,000–100,000, moderate; 100,000–125,000, good; 125,000–150,000, strong; >150,000, very strong. Immunoblot results were graded by the color intensity of the dots with binding affinity increases with the color intensity of the dots. Western blots results were graded by both color intensity and the thickness of the bands with binding affinity increase with the color intensity and the thickness of the bands.
(DOCX)

S1 Fig Gel images of total serum protein profiles and Western blot results of species tested with protein A/G, protein L and anti-kangaroo antibody. (DOCX)

S1 Raw images (PDF)
