# Supplementary material for: Towards a pan marsupial sero-immunological tool in the demanding field of wildlife serology: Marsupial immunoglobulin-binding capability with protein A/G, protein L and anti-kangaroo antibody
Source: PLoS One. 2023 Dec 14;18(12):e0295820. doi: 10.1371/journal.pone.0295820 (PMC10721001; doi:10.1371/journal.pone.0295820)
Supplement: S1 Table — -, No binding; +, weak binding; ++, moderate; +++, good binding; ++++, strong binding; +++++, very strong binding. Note: Reciprocal of the ELISA OD values were graded as following <50, no binding; 50–75,000, weak; 75,000–100,000, moderate; 100,000–125,000, good; 125,000–150,000, strong; >150,000, very strong. Immunoblot results were graded by the color intensity of the dots with binding affinity increases with the color intensity of the dots. Western blots results were graded by both color intensity and the thickness of the bands with binding affinity increase with the color intensity and the thickness of the bands. (DOCX) [file pone.0295820.s001.docx]

| **Order** | **Family** | **Species name** | **Common name** | **Immunoblot** | | | **ELISA** | | | **Western blot** | | |
| --- | --- | --- | --- | --- | --- | --- | --- | --- | --- | --- | --- | --- |
|  |  |  |  | **Protein A/G** | **Protein L** | **Anti-kangaroo antibody** | **Protein A/G** | **Protein L** | **Anti-kangaroo antibody** | **Protein A/G** | **Protein L** | **Anti-kangaroo antibody** |
| Diprotodontia | Macropodidae | *Macropus dorsalis* | Black-striped wallaby | +++ | +++ | +++++ | +++ | +++ | +++++ | ++++ | +++ | +++++ |
| Diprotodontia | Macropodidae | *Macropus parma* | Parma wallaby | ++++ | +++ | +++++ | ++++ | +++ | +++++ | ++++ | ++++ | +++++ |
| Diprotodontia | Macropodidae | *Macropus parryi* | Whiptail wallaby | ++++ | ++++ | +++++ | ++++ | ++++ | +++++ | +++++ | ++++ | +++++ |
| Diprotodontia | Macropodidae | *Petrogale lateralis* | Black-flanked rock-wallaby | ++++ | +++ | +++++ | ++++ | +++ | +++++ | ++++ | +++ | +++++ |
| Diprotodontia | Macropodidae | *Petrogale penicillata* | Brush-tailed rock-wallaby | ++++ | ++++ | +++++ | ++++ | +++ | +++++ | +++ | ++++ | +++++ |
| Diprotodontia | Macropodidae | *Petrogale xanthopus xanthopus* | Yellow-footed Rock-wallaby | +++ | +++ | +++++ | ++++ | ++ | +++++ | +++ | +++ | +++++ |
| Diprotodontia | Macropodidae | *Macropus rufogriseus* | Red-necked wallaby | +++ | +++ | +++++ | ++++ | +++ | +++++ | +++ | +++ | +++++ |
| Diprotodontia | Macropodidae | *Macropus agilis* | Agile wallaby | ++++ | ++++ | +++++ | ++++ | +++ | +++++ | +++++ | +++++ | +++++ |
| Diprotodontia | Macropodidae | *Macropus giganteus* | Eastern grey kangaroo | ++++ | ++++ | +++++ | ++++ | +++ | +++++ | +++ | ++++ | +++++ |
| Diprotodontia | Macropodidae | *Macropus rufus* | Red kangaroo | ++++ | ++ | +++++ | ++++ | ++ | +++++ | ++++ | ++++ | +++++ |
| Diprotodontia | Macropodidae | *Macropus fuliginosus fuliginosus* | Western grey kangaroo | ++++ | +++ | +++++ | ++++ | +++ | +++++ | ++++ | +++ | +++++ |
| Diprotodontia | Macropodidae | *Macropus antilopinus* | Antilopine kangaroo | ++++ | +++ | +++++ | ++++ | +++ | +++++ | ++++ | ++++ | +++++ |
| Diprotodontia | Macropodidae | *Dendrolagus goodfellowi* | Goodfellow's tree-kangaroo | ++ | +++ | +++++ | ++ | +++ | +++++ | +++ | ++++ | +++++ |
| Diprotodontia | Macropodidae | *Macropus robustus erubescens* | Common Wallaroo | +++ | +++ | +++++ | +++ | +++ | +++++ | ++++ | ++++ | +++++ |
| Diprotodontia | Macropodidae | *Dorcopsis luctuosa* | Gray dorcopsis | ++++ | ++++ | +++++ | +++ | ++++ | +++++ | ++++ | ++++ | +++++ |
| Diprotodontia | Potoroidae | *Bettongia penicillata ogilbyi* | Woylie/brush-tailed bettong | +++ | ++++ | +++++ | ++ | ++++ | +++++ | +++++ | +++++ | +++++ |
| Diprotodontia | Potoroidae | *Bettongia lesueur* | Burrowing bettong | + | +++ | +++++ | + | +++ | +++++ | - | ++ | +++++ |
| Diprotodontia | Potoroidae | *Potorous longipes* | Long-footed potoroo | ++++ | ++++ | +++++ | ++++ | ++++ | +++++ | +++++ | ++++ | +++++ |
| Diprotodontia | Potoroidae | *Potorous tridactylus tridactylus* | Long-nosed Potoroo | ++++ | ++++ | +++++ | ++++ | +++ | +++++ | +++ | +++ | +++++ |
| Diprotodontia | Petauridae | *Gymnobelideus leadbeateri* | Leadbeater's possum | + | +++ | +++++ | + | ++ | +++++ | - | + | +++++ |
| Diprotodontia | Petauridae | *Petaurus breviceps breviceps* | Sugar glider | +++ | + | +++ | +++ | + | +++++ | - | - | + |
| Diprotodontia | Phalangeridae | *Trichosurus vulpecula* | Common brushtail possum | +++ | +++ | ++++ | +++ | +++ | +++++ | + | + | +++ |
| Diprotodontia | Phalangeridae | *Trichosurus cunninghami* | Mountain brushtail possum | + | +++ | ++++ | ++ | +++ | +++++ | + | ++ | +++ |
| Diprotodontia | Vombatidae | *Lasiorhinus latifrons* | Southern hairy-nosed wombat | - | ++ | +++++ | - | + | +++++ | - | ++ | +++++ |
| Diprotodontia | Pseudocheiridae | *Pseudocheirus peregrinus peregrinus* | Common ringtail possum | -/+ | +++ | +++++ | - | + | +++++ | - | -/+ | +++++ |
| Diprotodontia | Phascolarctidae | *Phascolarctos cinereus* | Koala | - | +++ | +++++ | - | ++ | +++++ | - | + | +++++ |
| Peramelemorphia | Peramelidae | *Perameles gunnii* | Eastern barred bandicoot | - | +++ | +++++ | - | ++ | +++++ | - | + | +++++ |
| Peramelemorphia | Peramelidae | *Isoodon obesulus* | Southern brown bandicoot | - | ++++ | +++++ | - | ++ | +++++ | - | + | +++++ |
| Peramelemorphia | Thylacomyidae | *Macrotis lagotis* | Greater bilby | - | -/+ | +++++ | - | - | +++++ | - | - | +++++ |
| Dasyuromorphia | Dasyuridae | *Dasyurus viverrinus* | Eastern quoll | ++++ | +++ | +++++ | ++ | + | +++++ | - | - | +++++ |
| Dasyuromorphia | Dasyuridae | *Dasyurus hallucatus* | Northern quoll | ++++ | +++ | +++++ | + | - | +++++ | - | - | +++++ |
| Dasyuromorphia | Dasyuridae | *Sarcophilus harrisii* | Tasmanian devil | ++++ | ++++ | +++++ | +++ | +++ | +++++ | + | ++ | +++++ |
| Carnivora | Felidae | *Felis catus* | Cat | +++++ | ++++ | -/+ | ++++ | +++ | - | +++++ | ++++ | + |
| Artiodactyla | Bovidae | *Bos taurus* | Cattle | +++++ | + | - | ++++ | - | - | +++++ | +++ | + |
| Galliformes | Phasianidae | *Gallus gallus* | Chicken | - | - | - | - | - | - | - | - | - |
| Rodentia | Muridae | *Mus musculus* | Mouse | ++ | ++++ | - | + | +++ | - | ++ | ++ | -/+ |
| Perissodactyla | Equidae | *Equus ferus* | Horse | ++++ | ++ | - | +++ | - | - | +++++ | ++ | - |

**Supplementary table 1:** Comparison of marsupial immunoglobulin binding affinity to protein A/G, protein L and polyclonal anti-kangaroo antibody using immunoblot, enzyme linked immunosorbent assay (ELISA) and Western blot.

-, No binding; +, weak binding; ++, moderate; +++, good binding; ++++, strong binding; +++++, very strong binding

Note: Reciprocal of the ELISA OD values were graded as following <50, no binding; 50-75,000, weak; 75,000 - 100,000, moderate; 100,000-125,000, good; 125,000 - 150,000, strong; >150,000, very strong. Immunoblot results were graded by the color intensity of the dots with binding affinity increases with the color intensity of the dots. Western blots results were graded by both color intensity and the thickness of the bands with binding affinity increase with the color intensity and the thickness of the bands.
