# Supplementary material for: Towards a pan marsupial sero-immunological tool in the demanding field of wildlife serology: Marsupial immunoglobulin-binding capability with protein A/G, protein L and anti-kangaroo antibody
Source: PLoS One. 2023 Dec 14;18(12):e0295820. doi: 10.1371/journal.pone.0295820 (PMC10721001; doi:10.1371/journal.pone.0295820)
Supplement: S1 Fig — (DOCX) [file pone.0295820.s002.docx]

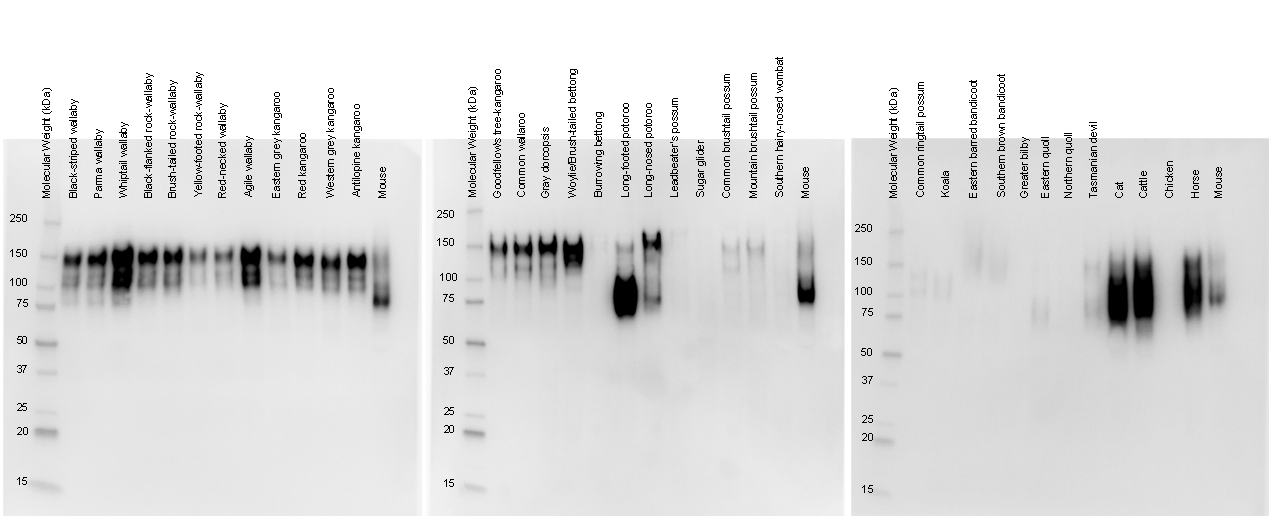

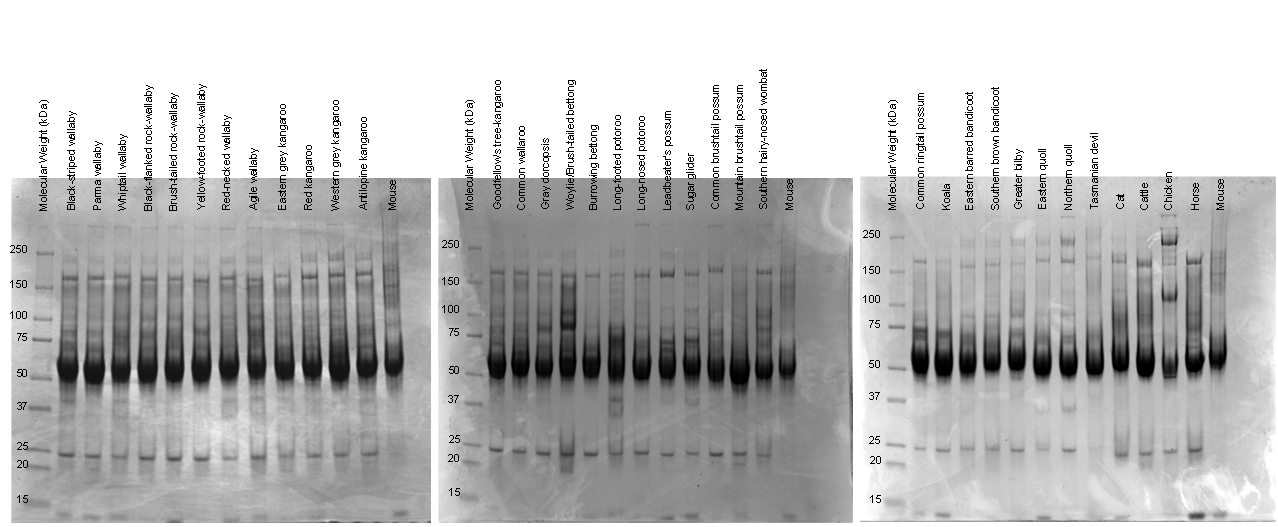


Western blot results of Australian marsupial sera with protein A/G (1:5000). 10ul of 1:25 diluted serum sample from each species were loaded into 4-15 % Mini PROTEAN TGX precast gel (BioRad) and ran for 72 minutes at 120V. DNA ladder used was Precision Plus Protein WesternC Standards 10 - 250 kD (BioRad). Following transfer of proteins, PVDF membranes were blocked with 5% skim milk powder in PBS overnight at 4°C . Following incubation with protein A/G, finally membranes were incubated with ECL and visualized using ChemiDoc MP Imaging system (BioRad).

Gel images of total serum protein profiles of Australian marsupials. 10ul of 1:25 diluted serum sample from each species were loaded into 4-15 % Mini PROTEAN TGX precast gel (BioRad) and ran for 72 minutes at 120V. DNA ladder used was Precision Plus Protein WesternC Standards 10 - 250 kD (BioRad). Gels were stained with Coomassie blue stain before visualization using ChemiDoc MP Imaging system (BioRad).


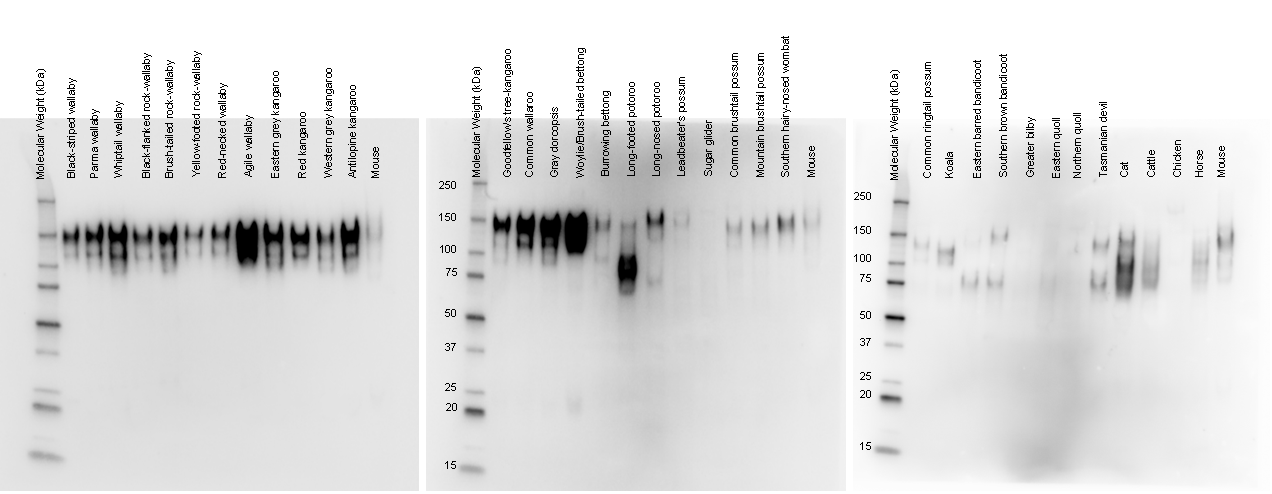


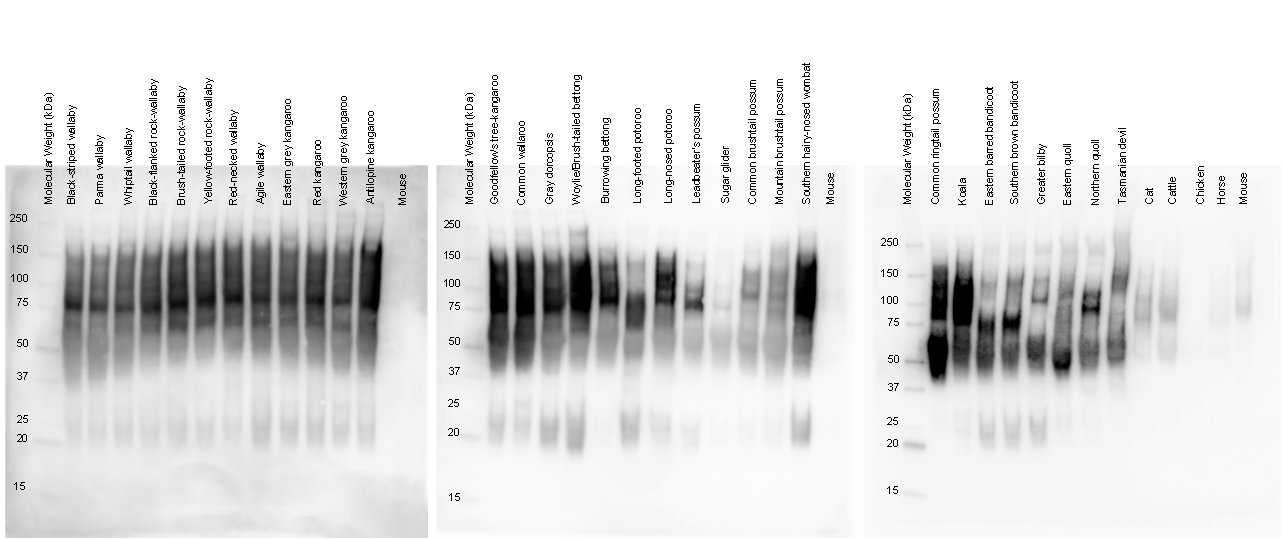


Western blot results of Australian marsupial sera with protein L (1:4000). 10ul of 1:25 diluted serum sample from each species were loaded into 4-15 % Mini PROTEAN TGX precast gel (BioRad) and ran for 72 minutes at 120V. DNA ladder used was Precision Plus Protein WesternC Standards 10 - 250 kD (BioRad). Following transfer of proteins, PVDF membranes were blocked with 5% skim milk powder in PBS overnight at 4°C. Following incubation with protein L, finally membranes were incubated with ECL and visualized using ChemiDoc MP Imaging system (BioRad).

Western blot results of Australian marsupial sera with rabbit polyclonal anti-kangaroo whole serum antibody (1:4000) and goat anti-rabbit IgG-antibody (1:5000). 10ul of 1:25 diluted serum sample from each species were loaded into 4-15 % Mini PROTEAN TGX precast gel (BioRad) and ran for 72 minutes at 120V. DNA ladder used was Precision Plus Protein WesternC Standards 10 - 250 kD (BioRad). Following transfer of proteins, PVDF membranes were blocked with 5% skim milk powder in PBS overnight at 4°C . Following incubation with rabbit polyclonal anti-kangaroo antibody and goat anti-rabbit IgG antibody, finally membranes were incubated with ECL and visualized using ChemiDoc MP Imaging system (BioRad).
