# Supplementary material for: Towards a pan marsupial sero-immunological tool in the demanding field of wildlife serology: Marsupial immunoglobulin-binding capability with protein A/G, protein L and anti-kangaroo antibody
Source: PLoS One. 2023 Dec 14;18(12):e0295820. doi: 10.1371/journal.pone.0295820 (PMC10721001; doi:10.1371/journal.pone.0295820)
Supplement: S1 Raw images — (PDF) [file pone.0295820.s003.pdf]

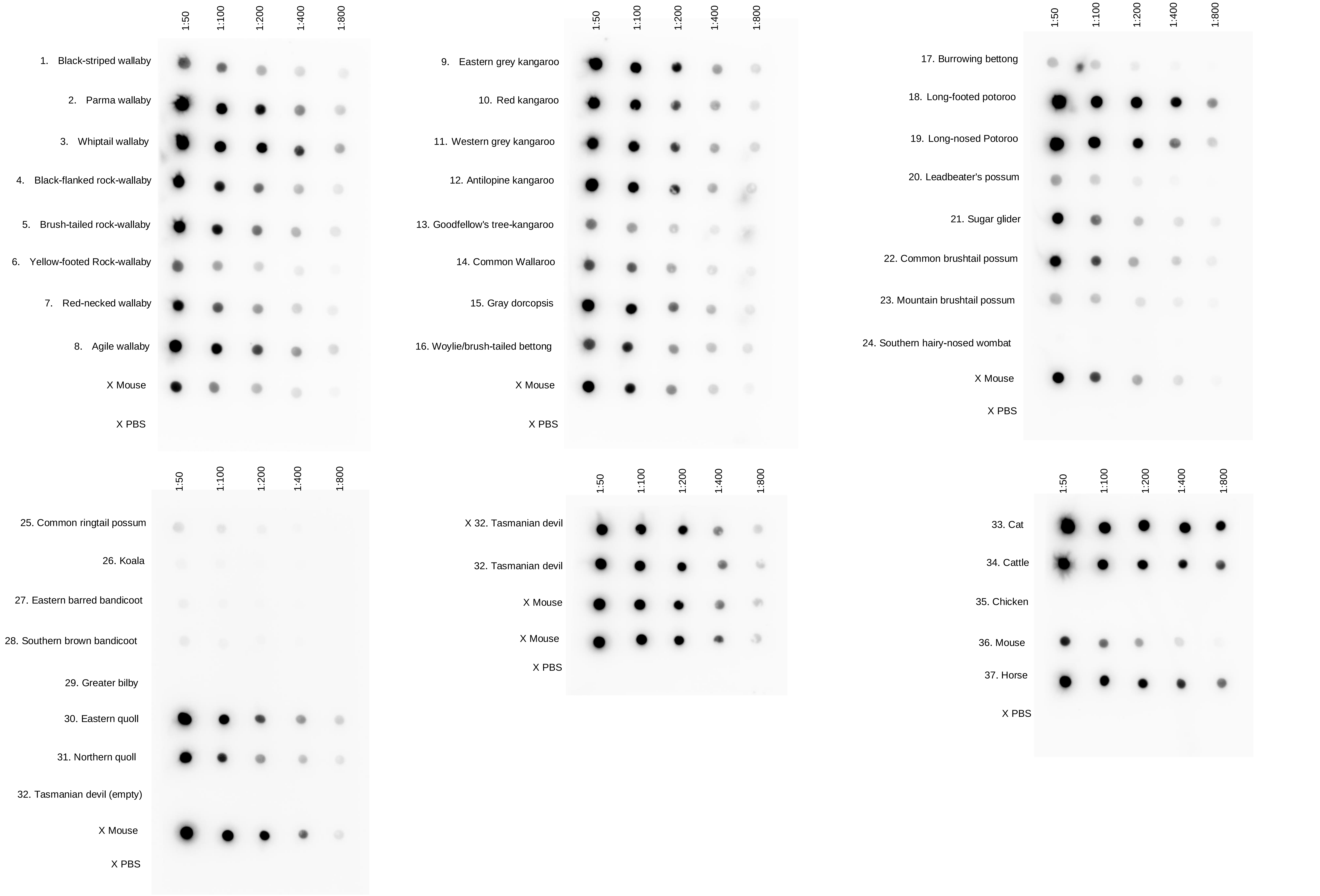

Original and uncropped immunoblot images showing strength of binding to sera of a range of metatherian (marsupial) and eutherian (placental) mammals with protein A/G (1:5000) with darker color blots representing stronger binding. Serum dilutions are indicated in the top row of each image. Immunoblot membranes were visualized using ChemiDoc MP Imaging system (Bio-Rad).

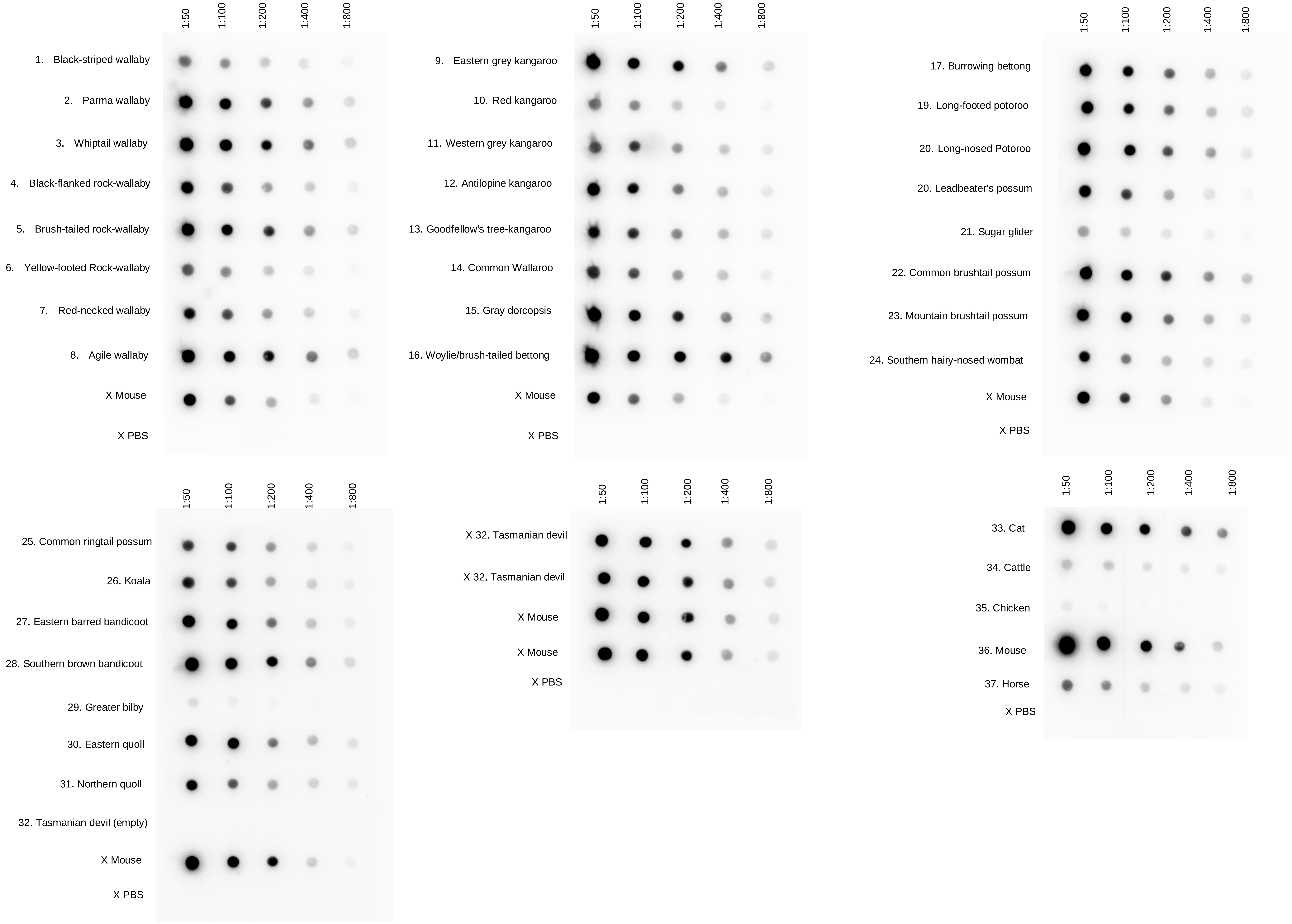

Original and uncropped immunoblot images showing strength of binding to sera of a range of metatherian (marsupial) and eutherian (placental) mammals with protein L (1:4000) with darker color blots representing stronger binding. Serum dilutions are indicated in the top row of each image. Immunoblot membranes were visualized using ChemiDoc MP Imaging system (Bio-Rad).

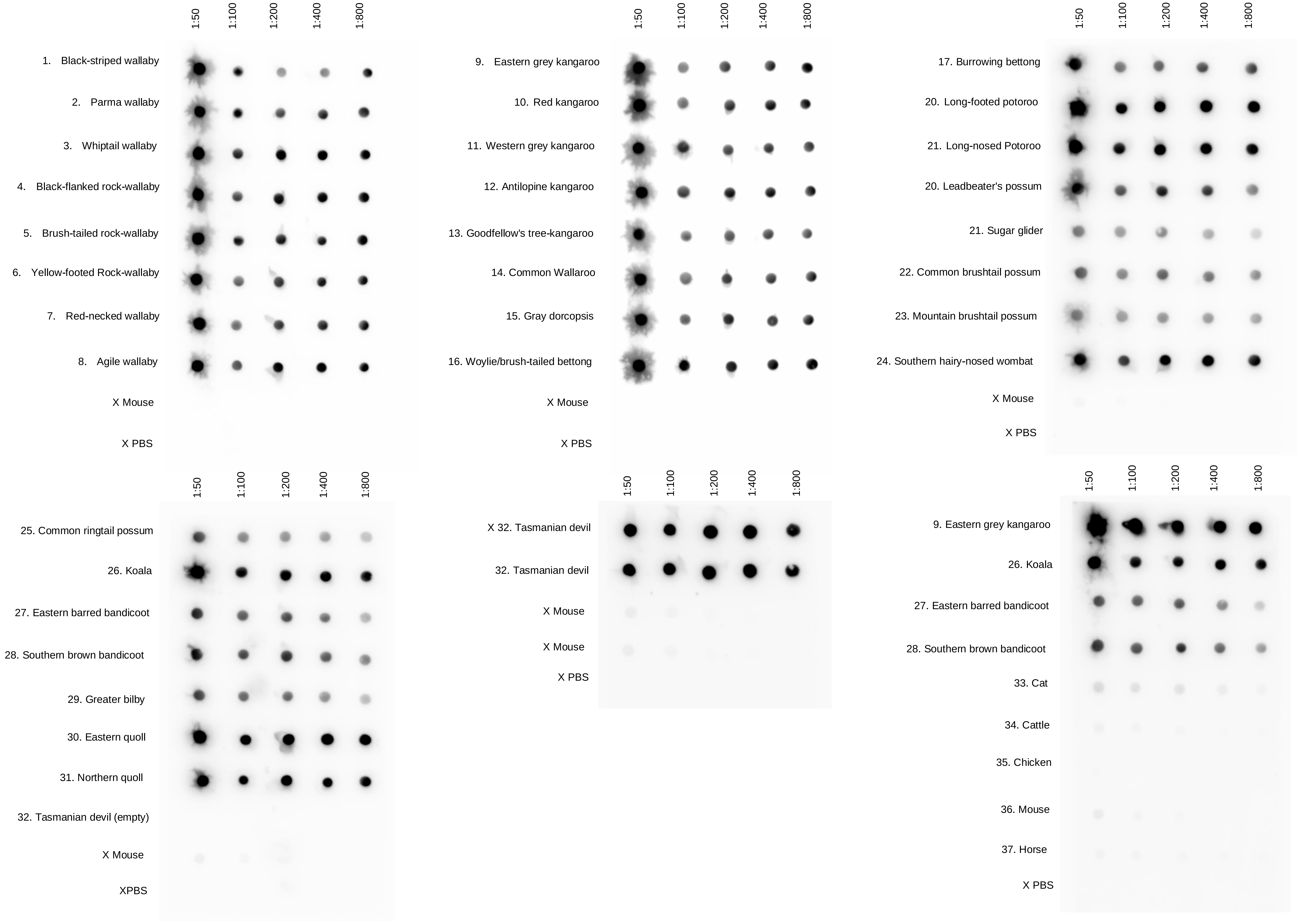

Original and uncropped immunoblot images showing strength of binding to sera of a range of metatherian (marsupial) and eutherian (placental) mammals with anti-kangaroo antibody (1:4000) and goat anti-rabbit antibody (1:5000) with darker color blots representing stronger binding. Serum dilutions are indicated in the top row of each image. Immunoblot membranes were visualized using ChemiDoc MP Imaging system (Bio-Rad).

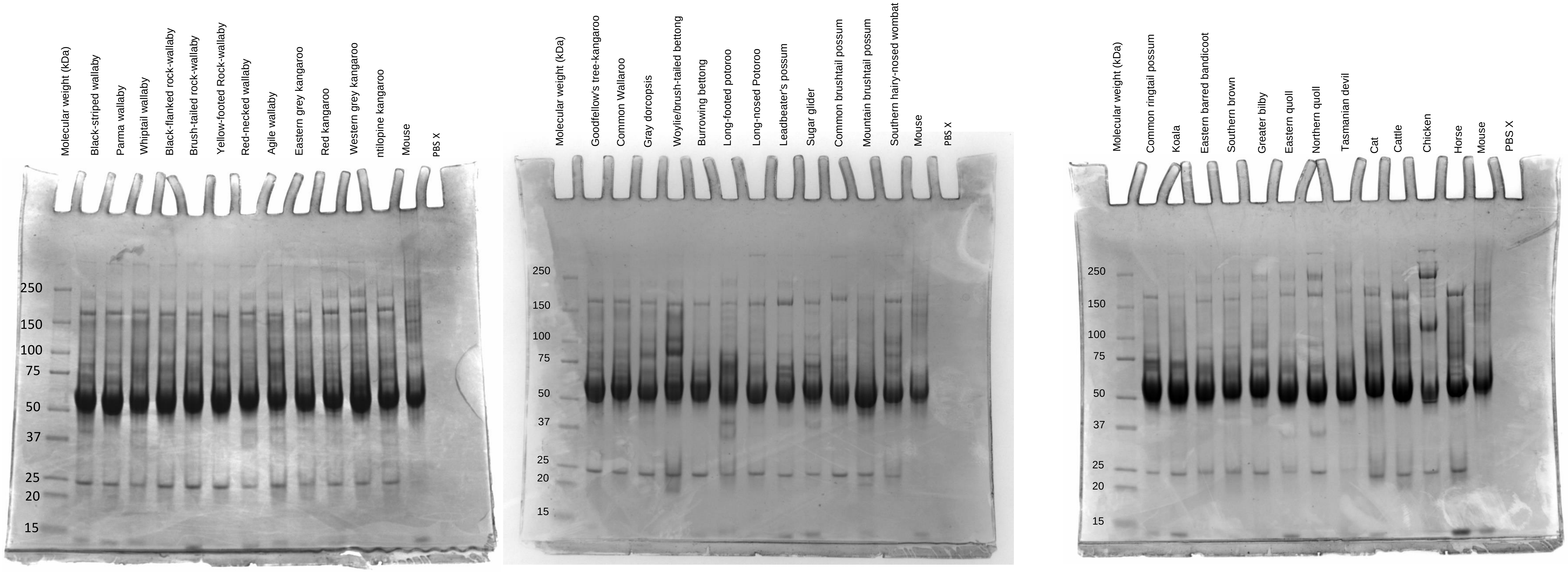

Original and uncropped gel images of total serum protein profiles of Australian marsupials. 10ul of 1:25 diluted serum sample from each species were loaded into 4-15 % Mini PROTEAN TGX precast gel (BioRad) and ran for 72 minutes at 120V. DNA ladder used was Precision Plus Protein WesternC Standards 10 - 250 kD (BioRad). Gels were stained with Coomassie blue stain before visualization using ChemiDoc MP Imaging system (Bio-Rad).
